# No influence of the indication of freeze-all strategy on subsequent outcome to frozen-thawed embryo transfer cycle

**Published:** 2018-06

**Authors:** T Masschaele, F Vandekerckhove, P De Sutter, J Gerris

**Affiliations:** AZ West, Ieperse Steenweg 100, 8630 Veurne; Center for Reproductive Medicine, University Hospital Ghent, Belgium. Corneel Heymanslaan 10, 9000 Gent, Belgium

**Keywords:** Freeze-all, blastocyst transfer, IVF, frozen embryo transfer, progesterone elevation

## Abstract

**Background:**

Freezing all embryos generated during an IVF/ICSI attempt is used increasingly as a strategy to optimize results. We investigated whether we could find differences in outcome between subpopulations of patients undergoing the so-called “freeze all” procedure.

**Methods:**

Non-interventional, observational, retrospective study of 131 freeze-all cycles performed between July 2015 and December 2016 at the University Hospital of Ghent (Belgium). Freeze-all indications were categorized in 4 groups: group 1, high progesterone level (PE) on the day of hCG administration defined as >1.5 ng/ml (n= 50); group 2, risk of Ovarian Hyperstimulation Syndrome (OHSS) (n=38); group 3, partner donation in lesbian couples (n=23) and group 4, a miscellany of other reasons (n=20). Clinical pregnancy with fetal heart beat after the first thawed embryo transfer (ET) after ovum pick-up and cumulative clinical pregnancy rate per attempt were used as primary outcome variables.

**Results:**

Clinical pregnancy rate (CPR) with fetal heart beat in the first thawed ET and cumulative clinical pregnancy rate per cycle (CCPR) were not statistically different between the four groups. In the group of PE a cumulative clinical pregnancy rate was observed of 40,5%, this in comparison to the 3 other groups involving risk of OHSS (66,7%), partner donation (61,1%) and other reasons (57,1%). More rFSH was used in the group with Progesterone elevation (PE) (P=0.04), as described earlier in the literature.

**Conclusion:**

Our findings indicate comparable (cumulative) clinical pregnancy rates per attempt between the four groups of freeze-all indications.

## Introduction

Recent technical improvements in cryopreservation have led to increased chances of embryo survival after thawing and subsequently increased pregnancy rates (PR) per frozen-thawed embryo transfer (FET) (Wong et al., 2014). Recent studies comparing ongoing pregnancy rates between FET with fresh ET show even statistically higher ongoing pregnancy rates in FET than in fresh ET (52% vs 45,3%) (Wang et al., 2017).

Several reasons justify the employment of the freeze-all strategy e.g. risk of OHSS (as defined elsewhere), serum progesterone elevation (PE) on the day of hCG administration (defined as >1.5 ng/ ml), lesbian between-couple partner oocyte donation and a miscellany of various other reasons (personal choice, endometrial problems, thyroid dysfunction).

It has already been demonstrated that the prevalence of OHSS is lower after a freeze-all strategy as compared to a conventional IVF/ICSI strategy (Wong et al., 2017).

The question whether the presence of increased serum progesterone (P) levels on the day of hCG administration is associated with the ongoing pregnancy rate (PR) remains subject to debate.

Bosch et al. (2010) reported that an elevated serum P concentration on the day of hCG administration (>1, 5 ng/ml) was associated with a lower ongoing PR. This value represents the critical threshold level above which there is a negative impact on implantation. A meta- analysis of over 60,000 IVF cycles concluded that, in fresh IVF attempts, elevation of serum progesterone >1,5 ng/ml is associated with a decreased PR (Venetis et al., 2013).

The detrimental effect of PE is thought to be exerted upon the endometrium. Elevated premature progesterone rise is likely to lead to embryo/ endometrial asynchrony, reducing the probability of implantation (Van Vaerenbergh et al., 2011; Labarta et al., 2011). Recently, two retrospective analyses observed a significant reduction in top quality embryo rate in relation to increasing P levels, suggesting a direct detrimental effect on the oocyte as well (Huang et al., 2016; Vanni et al., 2017).

The aim of the present study was to examine whether there is a difference in clinical outcome of freeze-all attempts between patient groups with different indications for freezing all embryos.

## Materials and methods

### Study design

We executed a non-interventional, observational, retrospective study carried out at the University Hospital of Ghent, Belgium. During the period July 2015 to December 2016, 131 freeze-all cycles were included in this study.

We identified four clinical groups in which we decided to freeze all embryos: group 1, PE on the day of hCG administration; group 2, elevated risk of OHSS; group 3, between-couple partner oocyte donation in lesbian couples and group 4, a miscellany of other reasons. PE on the day of hCG administration was defined as >1,5 ng/ml which is most frequently used cut-off in literature (Bosch et al., 2010). Patients with a risk of OHSS were defined by an estradiol > 3500 pg/ml with the use of urinary FSH or >2500 pg/ml with the use of recombinant FSH and/or ≥20 follicles ≥ 10 mm on day of hCG administration.

A miscellany of other reasons includes endometrial polyps, oncological reasons, severe thyroid dysfunction, PID (pelvis inflammatory disease), errors in luteal medication application or choice of the patient.

For not biasing our demographics only the first cycle was included of patients who underwent more than one freeze-all procedure. We included the women who had 2 different reasons for freeze- all transfer (avoid OHSS and high P level) in the main group: the high P level (5 patients). Oocyte donation cycles (excluding between-couple oocyte donation) and cycles including pre-implantation genetic diagnosis were excluded.

### Ethical approval

Approval by the Ethics Committee was obtained at 10/03/2017 (B670201731700).

### Ovarian stimulation protocol

Three types of protocols were used for pituitary suppression: the short agonist either with or without combined oral contraceptive pill, the long agonist using a triptorelin depot and the antagonist protocol as defined elsewhere (Vandekerckhove et al., 2012).

**Figure 1:**
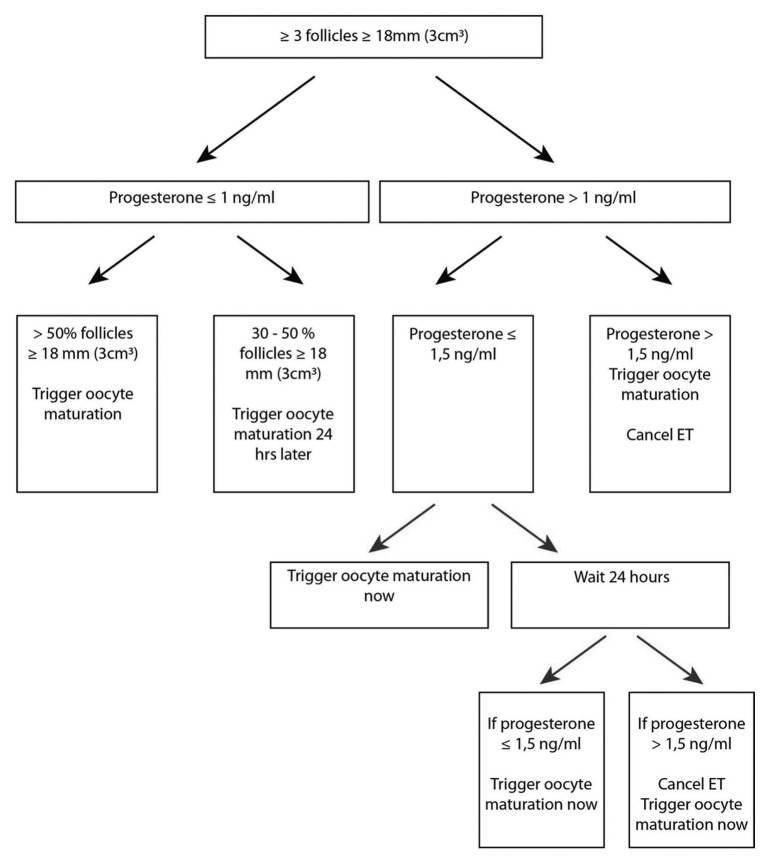
Maturation trigger decision algorithm

The decision algorithm regarding the moment of final oocyte maturation trigger used is shown in Figure 1.

Final oocyte maturation was induced with hCG (Pregnyl® 5000 IU; Human Chorionic Gonadotropin, Merck Sharp & Dohme, New Jersey, USA) subcutaneously. When patients were at risk of OHSS, oocyte maturation was induced using a GnRH agonist (Decapeptyl® 0.2 mg SC (Ipsen, Paris, France) if a GnRH antagonist had been used. Oocyte retrieval was conducted about 35 hours after the maturation trigger under alfentanil sedation and local vaginal lidocaine hydrochloride infiltration. Embryos were cryopreserved until day 5 by vitrification using a closed system with CBS-VIT high security straws (CryoBioSystel, L’aigle, France) and with dimethylsulphoxide- ethylene glycol-sucrose (DMSO-EG-sucrose) as cryoprotectant (Irvine Scientific Freeze Kit).

At least one cycle or one month following OPU elapsed before FET. In natural thawing cycles monitoring was performed using regular ultrasound scans and serum hormonal controls or urinary LH- tests at home. If the endometrium was ≥7 mm thick and triple lined and/or the LH-surge happened, we scheduled the ET 6 days after ovulation. In artificial thawing cycles the endometrial priming started on the second day of the menstrual cycle, using estradiol valerate orally 6 mg/d. After at least 7 days an ultrasound scan was performed. If the endometrium was ≥ 7 mm thick and triple lined, the FET was scheduled. In the latter protocol, the luteal support with vaginal micronized progesterone 600 mg/d (Utrogestan®, Besins Healthcare, Bangkok, Thailand) on daily basis was started 6 days before ET until the 12th week of gestation. If the endometrium was < 7 mm thick after endometrial priming, ET was cancelled.

Embryos were transferred under ultrasound guidance using a Cook-catheter (Curved Embryo Transfer Catheter, COOK® medical). The choice to transfer one or exceptionally two embryos was made by the clinician, depending on the patient’s age, embryo quality and cycle rank (Royal Decree 2003). Only blastocyst transfer of day 5 frozen embryos was performed.

### Hormone measurement

P and oestrogens (E2) were measured using an ECLIA, Modular E170 Roche system. Analytic sensitivity was 0,02 uU/ml for TSH (thyroid stimulating hormone), 0,03 ng/ml for AMH (anti- Müllerian hormone), 25 pg/ml for E2 and 0,15 ng/ml for P. Intra- and inter-assay precisions at the concentrations of most relevance to the study (expressed as coefficients of variation) were 2,3% for TSH, 3,5% for AMH, 3,2% for E2, 5,6% for P.

### Definition of Outcomes

The main outcome measure was clinical pregnancy rate (CPR) with fetal heartbeat (FHB), defined as a pregnancy diagnosed by ultrasonography showing at least one fetus with positive heart action. It also includes ectopic pregnancy (Zegers- Hochschild et al., 2009). The cumulative clinical pregnancy rate (CCPR) is defined as the number of clinical pregnancies with FHB resulting from one initiated or aspirated ART attempt until the first clinical pregnancy occurred.

### Statistical Analysis

Data were collected and analyzed in the statistical program SPSS version 24 (IBM SPSS Statistics 24.0, IBM Corp., Armonk, NY, USA). Categorical variables were compared using the Fisher’s exact test. Continuous data were compared using the Kruskal-Wallis test. A p-value <0,05 was considered to indicate significance (two-sided). Logistic regression analysis was performed. An odds ratio (OR) with 95% confidence interval (CI) was calculated.

## Results

Table I and II summarize the demographic characteristics, protocol characteristics and the different reasons for IVF in the four groups. In the group at risk of OHSS there are several findings indicating a better prognosis: the number of oocytes retrieved and E2 value at the day of hCG administration were significantly higher in the group with risk of OHSS than in the group of PE and the group of other reasons. Significantly more recombinant FSH was used in the group of PE than in the partner donation group (46% versus 13%, P= 0.008).

**Table I:** Baseline characteristics in the four groups who underwent freeze-all. Results are described as median (25th percentile – 75th percentile) or percentage (%).

	High Progesterone group (n=50)	Risk of OHSS group (n=38)	Partner donation (n=23)	Other reasons (n=20)	P value	All groups (n=131)
Age (years) (n=129)	33.9 (30.6-35.2)	33.4 (30.9-37.2)	33.6 (29.8-35.6)	33.2 (31.9-35.2)	0.976	33.5 (30.7-36.4)
BMI (n=108)	21.5 (20.2-23.7)	22.9 (20.1-24.9)	23.3 (21.7-26.2)	24.3 (20.7-26.6)	0.129	22.3 (20.5-25.2)
Duration infertility (years) (n=101)	3.7 (2.7-5.7)	4.2 (2.1-6.2)	0.6 (0.4-2.4)	3.8 (1.4-4.8)	<0.001^a,b,c^	3 (1.3-5.1)
Gravidity (n=130)	1 (0-1)	0 (0-1)	0 (0-0)	1 (0-1)	0.009^b,c^	0 (0-1)
Parity (n=130)	0 (0-0)	0 (0-0)	0 (0-0)	0 (0-1)	0.096	0 (0-1)
AMH (μl/dl) (n=122)	2.1 (1.3-3.7)	4.1 (2.4-5.6)	2.8 (1.7-4.5)	2.6 (1.7-3.3)	0.005^e^	2.8 (1.6-4.5)
TSH (mU/L) (n=108)	1.5 (1.0-1.8)	1.6 (1.1-1.9)	1.5 (0.6-2.1)	1.5 (1.2-2.0)	0.844	1.5 (1.0-1.9)
Cycle ranking (n=131)	2 (1-3)	2 (1-3)	1 (1-2)	2 (1-4)	0.004^c^	2 (1-3)
Smoking yes/no (n=102)	4 (10%)	3 (10%)	4 (22%)	0 (0%)	0.267	11 (8.4%)
Alcohol yes/no (n=75)	9 (32%)	10 (40%)	5 (38%)	5 (55%)	0.676	29 (22%)
Uterine fibroids (n=131)	0 (0%)	3 (8%)	1 (4%)	0 (0%)	0.127	4 (3.1%)
Uterine anomaly (n=131)	0 (0%)	1 (3%)	0 (0%)	0 (0%)	0.618	1 (0.8%)
Thyroid dysfunction (n=131)	7 (15%)	10 (26%)	2 (9%)	6 (30%)	0.155	25 (19%)
Immunologic factors/Thrombophilia (n= 131)	6 (12%)	5 (13%)	1 (4%)	2 (10%)	0.771	14 (10%)
PID in history (n= 131)	1 (2%)	1 (3%)	1 (4%)	0 (0%)	0.873	3 (2%)
Cardiovascular risk factors (n= 131)	1 (2%)	2 (5%)	0 (0%)	0 (0%)	0.650	3 (2%)
Abdominal surgery (n= 131)	7 (14%)	5 (13%)	3 (13%)	5 (25%)	0.641	20 (15%)
Reasons for IVF						
Male factor (n=131)	28 (56%)	22 (58%)	0 (0%)	11 (55%)	<0.001 ^a,b,c^	61 (47%)
Ovarian factor (n=131)	5 (10%)	5 (13%)	0 (0%)	2 (10%)	0.353	12 (9%)
Endometriosis (n=131)	8 (16%)	6 (16%)	0 (0%)	3 (15%)	0.175	17 (13%)
Tubal factor (n=131)	4 (8%)	3 (8%)	0 (0%)	0 (0%)	0.429	7 (5%)
Idiopathic (n=131)	9 (18%)	6 (16%)	0 (0%)	1 (5%)	0.088	16 (12%)
Oncologic reasons (n= 131)	2 (4%)	0 (0%)	0 (0%)	4 (20%)	0.005	6 (5%)

Pairwise comparisons (^a^ = partner donation versus risk of OHSS, ^b^ = partner donation versus other reasons, ^c^ = partner donation versus high progesterone, ^d^ = risk of OHSS versus other reasons, ^e^ = risk of OHSS versus high progesterone, ^f^ = other reasons versus high progesterone) Note: n= number of patients; BMI, body mass index; AMH: anti-müllerian hormone; TSH: thyroid- stimulating hormone; PID: pelvic inflammatory disease

**Table II:** Cycle characteristics in the 4 groups of patients who underwent freeze-all. Results are described as median (25th percentile – 75th percentile) or percentage (%).

	High Progesterone (n=50)	Risk of OHSS (n=38)	Partner donation (n=23)	A miscellany of other reasons (n=20)	P-value	All groups (n=131)
Recombinant gonadotropin (n=131)	23 (46%)	11 (29%)	3 (13%)	6 (30%)	0.038^c^	43 (32,8%)
Urinary gonadotropin (n=131)	28 (56%)	27 (71%)	20 (87%)	14 (70%)	0.061	89 (68%)
Agonist short (n=131)	34 (68%)	16 (42%)	13 (57%)	15 (75%)	0.042	78 (60%)
Agonist long (n=131)	3 (6%)	4 (11%)	1 (4%)	2 (10%)	0.752	10 (8%)
Antagonist (n=131)	13 (26%)	18 (47%)	9 (39%)	3 (15%)	0.046	46 (33%)
Progesterone (μg/L) on day hCG-trigger (n=127)	2.1 (1.7-2.4)	0.7 (0.5-0.9)	0.8 (0.4-1.0)	0.8 (0.6-1.1)	<0.001^c,e,f^	1.1 (0.7-1.8)
Estradiol (ng/L) on day hCG-trigger (n=125)	2020 (1130-2830)	3089 (1940-3920)	1830 (1120-2660)	1570 (1270-2080)	0.004^d,e^	2030 (1285-3091)
Number oocytes at OPU (n=131)	13 (10-19)	19 (13-24)	15 (10-17)	12 (7-15)	<0.001^d,e^	14 (10-19)
ICSI (n=131)	44 (88%)	34 (90%)	23 (100%)	18 (90%)	0.400	119 (91%)

Pairwise comparisons (^a^ = partner donation versus risk of OHSS, ^b^ = partner donation versus other reasons, ^c^ = partner donation versus high progesterone, ^d^ = risk of OHSS versus other reasons, ^e^ = risk of OHSS versus high progesterone, ^f^ = other reasons versus high progesterone).

Clinical outcome was co-determined by serum AMH, serum progesterone, rank of stimulation attempt, duration of infertility, peak level of serum E2 and the number of oocytes retrieved. We have chosen the most important covariate, level of serum E2 on day of hCG administration, for correcting our results. Clinical outcomes are described in Table III.

**Table III:** Outcome of cryo-thaw transfers in the 4 groups of patients who underwent freeze-all. Odds Ratio (OR) (Confidence Interval 95% lower - upper).

	High Progesterone (n=50)	Risk of OHSS (n=38)	Partner donation (n=23)	A miscellany of other reasons (n=20)	P-value
Frozen embryo transfer occurred (yes/no) (n=131)	84 % (n=42)	86,8% (n=33)	78,3% (n=18)	70% (n=14)	0,412^a^
	OR 1,35 (0,35-5,19)	OR 2,03 (0,42-9,79)	Reference group	OR 0,61 (0,14-2,71)	0,461^b^
Clinical pregnancy rate after first cryothaw transfer (%) (n=107)	21,4% (n=9)	27,3% (n=9)	38,9% (n=7)	35,7% (n=5)	0,472^a^
	OR 0,43 (0,13-1,43)	OR 0,72 (0,20– 2.57)	Reference group	OR 0.86 (0,20-3.66)	0,513^b^
Cumulative clinical pregnancy rate (all frozen) (%) (n=107)	40.5 % (n=17)	66,7% (n=22)	61.1 % (n=11)	57,1% (n=8)	0,128^a^
	OR 0,43 (0,14-1,35)	OR 1,27 (0,37 -4,40)	Reference group	OR 0.84 (0,20-3.48)	0,169^b^

Note: ^a^: Fisher’s exact test ^b^: Logistic regression analysis, results adjusted for estradiol value on day of hCG trigger.

In our study the CCPR was 54.2% per cycle in the total freeze-all group. Although a lower clinical PR was observed in the PE group (40.5%) compared with the other groups, the difference is not statistically significant. Almost all blastocyst transfers were performed as single embryo transfer, only 6 ETs were double embryo transfer (DET). The distribution of double embryo transfers did not differ between the study groups. As DET was only performed in 6 cases with low embryo quality, our results were not influenced by our transfer policy. One twin pregnancy was observed.

## Discussion

Recently it has been suggested that the rise in progesterone does not only has its detrimental effect on the endometrium but also on the embryo. Our results also suggest this, although this difference is not significant. In the group of PE a cumulative clinical pregnancy rate was observed of 40,5%, compared to the three other groups: risk of OHSS (66,7%, partner donation (61,1%) and other reasons (57,1%). This non-significant result could be explained by the low number of patients. Moreover we see a higher cumulative clinical pregnancy rate in the group at risk of OHSS and partner donation than in the group of PE and other reasons, though not statistically significant (64,7% versus 44,6%, P=0,050 adjusted for estradiol, P=0,052 if not adjusted for estradiol).

The current study is characterized by certain limitations that should be discussed. Although the effect of the most important confounder was controlled for in this analysis, the presence of residual bias in a retrospective study cannot be excluded. Another limitation is that we were unable to investigate live birth and perinatal outcomes. It’s hard to draw firm conclusions and larger numbers are needed to confirm this result.

PE during ovarian stimulation appears to originate either from the ovaries and/or the adrenals and there are several factors that can contribute to this rise. It is well documented that serum P levels at the time of hCG administration is significantly correlated with the magnitude of ovarian response to stimulation (Bosch et al., 2010, Kyrou et al., 2012; Griesinger et al., 2013; Venetis et al., 2013; Venetis et al., 2015). Indirectly as a result, a high serum E2 concentration on day of hCG administration (Kyrou et al., 2012, Bosch et al, 2010) is also associated with PE. Venetis et al. showed that basal serum progesterone (day 2) and a history of PE are significant predictors of late follicular progesterone elevation in GnRH antagonist cycles (Venetis et al., 2016).

The question arises, if PE has a detrimental effect on the endometrium and the oocyte, how can we avoid PE or manage it ? (Lawrenz et al., 2018).

In cycles with PE on the day of hCG administration, embryo cryopreservation and cancellation of fresh transfer might be an option (Venetis et al., 2013). Though this will only have its effect on the endometrium, not on the oocyte quality.

Significantly higher serum progesterone levels were observed in patients treated with recombinant FSH (recFSH) than those with highly purified menotrophin (HP-hMG) for controlled ovarian stimulation in GnRH antagonist cycles (Bosch et al., 2008) and GnRH agonist cycles (Andersen et al., 2006). Our results confirm these findings. HP-hMG contains FSH and hCG-driven LH-activity whereas recFSH contains only FSH. The hCG-driven LH activity in HP-hMG stimulation may offset the rise in progesterone by stimulating theca cell activity towards the catabolism of progesterone to androgens and, thereafter, metabolism to estrogens in granulosa cells. When PE occurs, it would be reasonable to stimulate the next cycle with an HP-hMG.

Other strategies such as mild stimulation protocols, and lowering the stimulation duration were suggested. However, the fact that the number of retrieved oocytes is associates with the probability of live birth (Sunkara et al., 2011), such a strategy should only be considered with patients that are at risk of PE.

Further research would be needed to shed more light on the effect of progesterone rise on oocyte and embryo quality.
